# Leachate from Municipal Waste Landfill and Its Natural Degradation—A Case Study of Zubří, Zlín Region

**DOI:** 10.3390/ijerph13090873

**Published:** 2016-09-01

**Authors:** Vojtěch Václavík, Ivana Ondrašiková, Tomáš Dvorský, Kateřina Černochová

**Affiliations:** 1 Institute of Environmental Engineering, Faculty of Mining and Geology, VSB-Technical University of Ostrava, 17. listopadu15, Ostrava 708 00, Czech Republic; tomas.dvorsky@vsb.cz (T.D.); katerina.cernochova.st@vsb.cz (K.Č.); 2 AZ GEO s.r.o., Ostrava, Masná 1493/8, Ostrava 702 00, Czech Republic; ondrasikova@azgeo.cz

**Keywords:** landfill, waste (seepage) water, inorganic and organic contaminants, long-term trends, biological pond

## Abstract

This work deals with the natural degradation of leachate from an old reclaimed landfill by means of a biological pond. Hamra is a municipal waste landfill with a limited formation of leachate, which has already been reclaimed. Leachate in this location is disposed of using natural biogeochemical method, and it is subsequently discharged into a surface stream. The main issue dealt with here is the long-term effectiveness of natural degradation of leachate and the limits of its use. The solutions of these fundamental questions took advantage of a database of analytical assessments collected during a long-term monitoring of the landfill site. The primary degradation trends and the long-term development have been revealed and described on the basis of these assessments. The main benefit of the biological pond is the dilution of the dominant contaminants, especially of inorganic character. In the case of ammonium ions, they show nitrification caused by their transition from the reduction into oxidizing environment. From a long term point of view, the disadvantage of natural degradation of leachate can be seen in the gradual reduction in efficiency due to the concentration of the substances or an undesired growth of water plants, which can be successfully eliminated, for example, by means of targeted aeration and by maintaining vegetation in the pond and its surroundings. The biological potential of the locality is very favorable and, despite its anthropogenic load, it creates a location with suitable living conditions for many water animals and plants. That is why it can be concluded that the efficiency of the natural biochemical cleaning elements can be considered as sufficient, taking into account the nature of the deposited waste, the quantity and quality of leachate, as well as the climate character of the locality.

## 1. Introduction

Municipal waste landfills represent a rich reservoir of a broad spectrum of contaminants of inorganic and organic nature, including a considerable group of specific compounds. The migration of these substances in water surface and rock ambient poses a major threat to the environment, especially to the quality of surface and groundwater [1]. There are studies dealing with the modelling of the migration of these substances in groundwater in Taiwan [2], along with efforts to influence the quality of leachate directly in situ using adsorbent materials based on zeolite and perlite [3]. Taking into account that landfilling is, and in the near future will continue to be, one of the most widespread methods of waste disposal, it is necessary to study this issue in detail. The negative impact of landfills on the environment is undeniable, as researches of groundwater pollution in Yemen or Egypt suggest [4,5].

However, by examining the individual landfills and processes taking place in landfills and their surroundings, we can observe and describe the basic principles of migration of substances in the rock and aqueous environment, the changes in physical and chemical conditions, and the aspects of the effects of these processes, such as the description of the flow of leachate in time and its understanding [6,7] or the monitoring of the composition of leachate over a longer period of time [8]. There are studies in the world focused on the disposal of landfill leachate using chemical, biological, physical, or combined methods [9,10,11,12,13]. One of the methods used for the cleaning and stabilization of leachate from landfills is the utilization of stabilization ponds, e.g., in Brazil, where intensified natural cleaning processes based on bacteria are used [14,15]. Biological pollution in leachate can also be removed by using various electrochemical processes combined with physical methods [16,17].

The acquired information can subsequently be further applied both in the construction of new landfills and in the operation of existing landfills, as well as during environmental disasters in chemical and other industrial plants.

The main issue of the planned study was a relevant evaluation of the effectiveness of the natural biological element in the disposal of landfill leachate with subsequent discharge into surface waters. This issue is presented on the example of a landfill in Zubří, where technical reasons made it impossible to collect landfill leachate separately and to dispose it, e.g., by means of a wastewater treatment plant. This is the reason why a biological pond was used as the cleaning element. The aspects of this leachate cleaning are evaluated on the basis of long-term series of analytical determinations of leachate and surface waters into which the purified waters are discharged.

## 2. Methodology of Work—Field Measurements, Analytical Methods, and Evaluation

Landfill monitoring systems represent an integral part of landfill operation. The scope of monitoring is based on the requirements of the landfill operator. Leachate (simple sample of landfill leachate) and surface water samples were taken statically (in spots) into the prescribed sample containers. After sampling, the samples susceptible to change (samples used for the determination of metals) were fixed in order to maintain the oxidation states using about 1 mL of concentrated HNO_3_. The samples were stored in a cool dark place, and they were immediately transported to a laboratory for processing. The samples were evaluated in hydro-chemical laboratory of Vodovody a kanalizace Vsetín a.s., Central Laboratory Company, which is an accredited laboratory. The methodology of the individual determinations was in line with the standard operating procedures in compliance with ČSN/EN/ISO standards. An overview of the methodology of laboratory determination, including the value of LOQ (the determination limit corresponding to the lowest value which allows a quantitative assessment) is presented in Table 1.

## 3. Information on Case Study Zubří

### 3.1. Landfill Characteristics and Location

Hamra landfill is located in the Czech Republic, on the west to south-west edge of Zubří municipality, in the Zlín Region, outside the built-up area of the city. Its northern border is a forest area, from the west and east side, the landfill is surrounded by agricultural areas, while in the south, it is situated near small wooded areas, a forest road and a residential building. The terrain is slightly steep; the altitude ranges from about 345 to 355 m above sea level.

**Figure 1:**
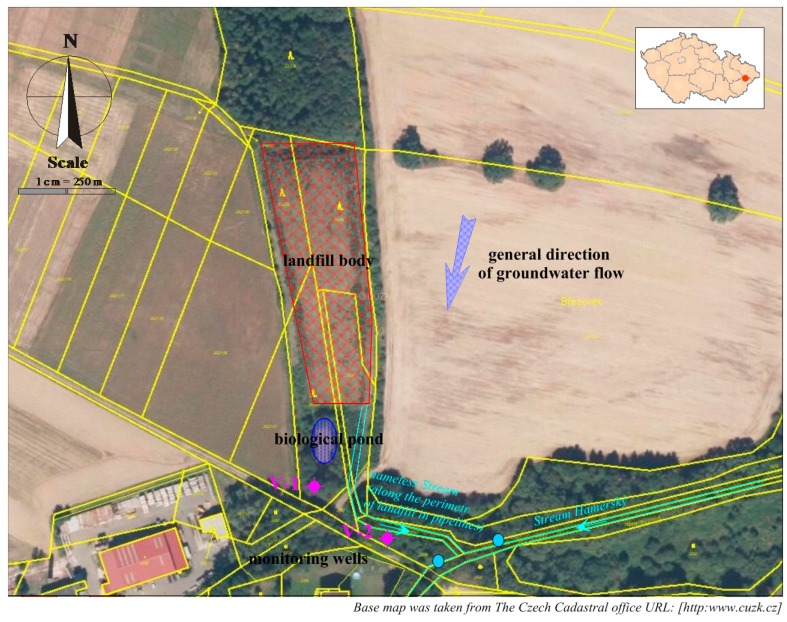
Description of the landfill situation with the monitoring system.

The landfill consists of artificially adapted terrain depression (erosion rill) of trapezoidal shape, with an area of 7436 m^2^. The landfill has currently been reclaimed and re-planted. This location was used for landfilling from 1990 to 1996, when mainly municipal waste (a total of about 30,000 m^3^) was deposited here. A building permit for the reclamation of the landfill had been issued in 2000, and it was completed in 2004. The landfill surface is levelled, compacted, and covered with fine-grained material. A PEHD foil, protected by geo-textile material on both sides, is applied on the levelled surface, as well as a drainage layer and soil layer with the thickness of up to 2 m. The surface is covered with tree plants. There is no degassing device in the landfill due to the nature and amount of waste. There is a bio-pond built in the landfill as part of the landfill technology for self-cleaning of leachate and rainwater. The bottom of the pond is covered with a foil, and the leachate from the landfill and rainwater from the peripheral ditches are discharged into this pond. There is an unnamed stream flowing through a pipeline below the landfill body, which leads to an already open stream bed below the landfill at the mixed water mouth from the biological pond. After about 50 m, the inflow leads into the Hamerský Stream. The Hamerský Stream flows into the Rožnovská Bečva River after 1 km. See Figure 1.

### 3.2. Geological and Hydro-Geological Characteristics of the Locality

**Figure 2:**
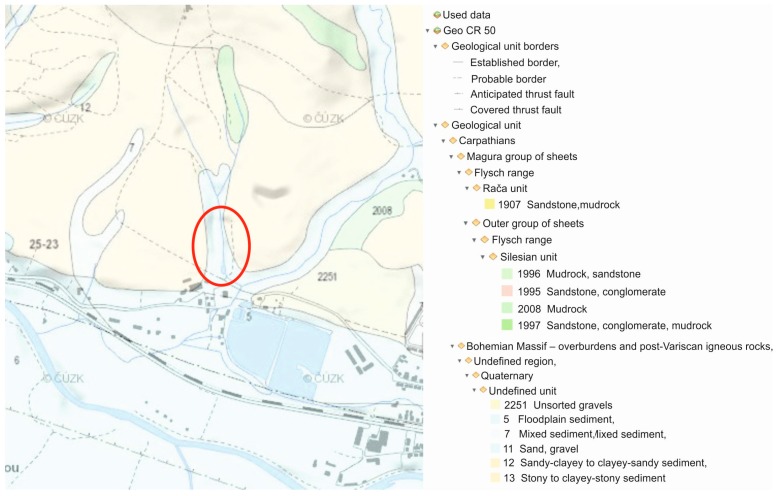
Geological situation of the wider surrounding area on a geological map of CR cut out (1:50,000) with an indicated area of interest.

In terms of the regional and geological division of the Czech Republic, the wider area of this locality belongs to the outer group of sheets of the Western Carpathian flysch range. The geological structure is formed by the Godula development of the Silesian Unit (near the contact with the Magura Group), whose rocks were settling in the Lower Cretaceous to Neogene periods, in which they went through orogeny and the termination of sediment settling. The direct bedrock of the locality consists of rocks of Istebna complex of strata (Upper Cretaceous to Paleogene), which make up a roughly rhythmic flysch of sandstones, conglomerates, inferior dark mudstones of psammitic-pelitic facies. The formation thickness is 400–1200 m. The sandstones are of quartz, arkose, and greywacke character, fine to coarse-grained, forming sequences which are separated from each other by thin layers of claystone. The conglomerates appear in gradationally stratified positions on the basis of sandstone benches. There are boulders of quartz, chalcedony, metamorphite, and igneous rocks in the conglomerates (see Figure 2).

According to the regional hydro-geological zoning of the Czech Republic, this area belongs to the group of flysch sediment zones, sub-zone of the base layer of no. 3221 flysch in the Bečva river-basin, occupying an area of 1291.56 km^2^. There is an unrestricted divisible collector within the zone, and it is tied to the environment of claystones and marlstones with free groundwater surface with predominant interstitial-fissure permeability. A continuous shallow quaternary aquifer is tied to interstitial permeable quaternary gravel sediments of the alluvial plain and clayey-rocky slope sediments. The permeability of soil varies considerably depending on the content of clay, sand, and gravel. The groundwater surface is shallowly locked below the terrain, it is free and its slope conforms to the terrain slope. The shallow quaternary aquifer groundwater drains to the Hamerský Stream, which forms the local drainage base. The subsurface flysch rocks, respectively their near-surface fissured and soft area represent, from the hydro-geological point of view, a regional insulator with increased permeability only in this near-surface soft zone. The transmissivity is low or even very low.

According to the hydrological division of the territory of the Czech Republic, the territorial localities are situated in the main river-basin of the Bečva River; in sub-basin of the fourth order with the number of 4-11-01-1162 the Hamerský Stream, with the river-basin area of 3.38 km^2^. The area is drained to the south or south-west direction into the Hamerský Stream valley, where it forms the local erosion basis. The Hamerský Stream flows into the Rožnovská Bečva River after 1 km (it is its right-side tributary).

Monitoring of ground and surface waters at the site is carried out on the following profiles

- Monitoring borehole V-1 and V-2 (groundwater)
- The inlet of the stream into the pipeline above the landfill, point A (surface water)
- Outlet out of the pipeline below the landfill, point B (surface-leachate water)
- Biological pond outlet, point C (surface water)
- Inlet into the Hamerský Stream, point D (surface water)

**Figure 3:**
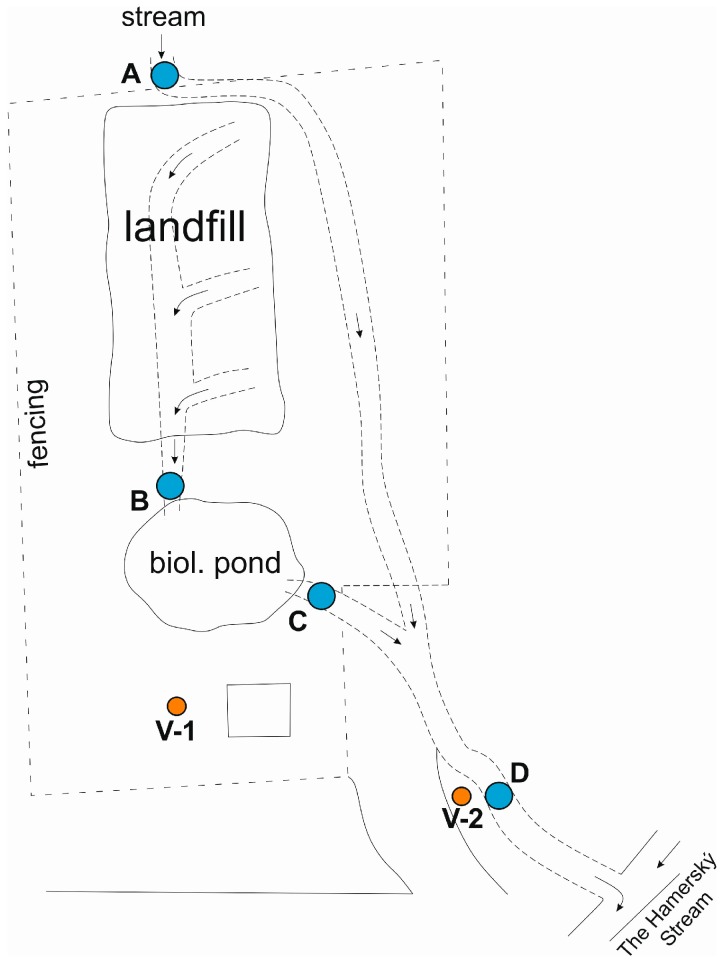
Schematic illustration of the locality with indicated water sampling points.

The range of determination is uniform and includes ammonium ions, nitrates, nitrites, pH, CODCr, conductivity, mercury, and HOI, in surface water also BOD5, PAH, and Cd. The sampling frequency is once a year (first half of the year—April). A schematic overview of the monitoring points is shown in Figure 3.

## 4. Results and Discussion

### 4.1. Groundwater Quality Assessment

**Figure 4:**
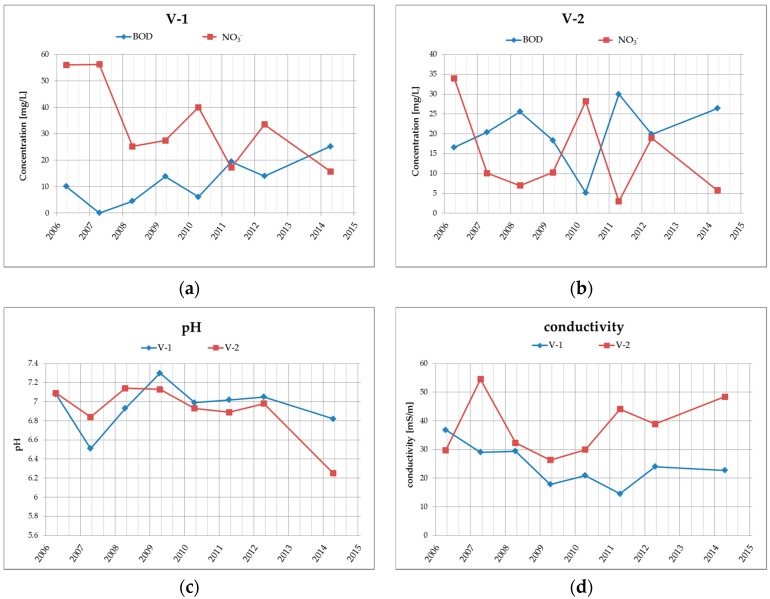
Graphical illustration of the long-term trend of (**a**) BOD and NO_3_^−^ in borehole V-1; (**b**) BOD and NO_3_^−^ in borehole V-2; (**c**) water reaction—pH in boreholes V-1 and V-2; (**d**) conductivity in boreholes V-1 and V-2.

Groundwater quality at the site is monitored in boreholes V-1 and V-2, which are located at the landfill drainage profile. The results from the years 2006–2014 (see Table 2 and Figure 4) clearly show that the groundwater in this area, respectively at the drainage profile, has medium-high specific conductivity (conductivity varies within the range of 15 to 55 mS/m) and neutral to very slightly acidic water reaction (pH is within the range of 6.25 to 7.3). Ammonium ions are found in higher levels in groundwater in the examined locations only occasionally with a maximum of 0.89 mg/L. From heavy metals, there are higher concentrations of only Hg, while the contents of other heavy metals, such as Pb, Zn, Cu, Cr, in the amounts exceeding the limits were not confirmed in the past.

Due to the fact that the leachate does not have higher mercury content (the maximum concentration of Hg in leachate in the last 8 years was 0.0002 mg/L see Table 3, sample B), the concentration of Hg in groundwater exceeding the limit cannot be evaluated in relation with the influence of the landfill. Increased concentrations occur very rarely, only in borehole V-1, which is why it can be assumed that the increased mercury content is more likely related to anthropogenic impacts, e.g., the impact of intensive agriculture (use of different mercury pesticides and inhibitors) or to fallout after the combustion of fossil fuels. Higher mercury content may also be related to increased natural background concentration of Hg in sediments of the Istebna formation.

### 4.2. Surface Water Quality Assessment

Surface water quality assessment was performed using the data series of monitoring results from the years of 2006 to 2014. A summary of selected results of surface water monitoring is shown in the following Table 3.

#### 4.2.1. Profile A—Unaffected Environment

The quality of surface water in profile A, which represents the unaffected environment, has been relatively stable since 2006, with low specific conductivity content (specific conductivity ranges up to 25 mS/m), the water reaction is neutral to slightly alkaline (pH = 7.1–7.8). The contents of ammonium ions and nitrates in increased concentrations occur only sporadically, from heavy metals, only Hg exceeds the detection limit with the maximum of 0.0002 mg/L. This section may be divided by subheadings. It should provide a concise and precise description of the experimental results, their interpretation as well as the experimental conclusions that can be drawn. The values of organic substances represented by the COD and BOD parameters, with maximum values of COD = 84 mg/L and BOD = 22 mg/L, have increased as well. PAHs are virtually absent in surface water in this profile, HOI oil products have been verified with a maximum value of 0.24 mg/L.

#### 4.2.2. Profile B—Leachate from the Landfill 

Leachate from the landfill has neutral to very slightly acidic reaction (pH = 6.48 to 7.3), a higher specific conductivity (max. specific conductivity was 105.7 mS/m). The contents of heavy metals, apart from Hg, were below the detection limit by means of a laboratory method (Cd, Pb), the content of mercury reaches a max. value of 0.0002 mg/L. The content of ammonium ions is usually up to 5 mg/L, with a rare maximum concentration of 12.32 mg/L. The contents of nitrites are low, up to 0.06 mg/L, the maximum nitrate content was 5.1 mg/L. The content of organic substances (permanganate index, BOD) reached a maximum of 43.8 mg/L in case of COD and 21.8 mg/L in case of BOD. The content of polyaromates reached a maximum of 680 mg/L, during the last five years, however, the maximum concentration value reached 180 mg/L. Oil products (HOI) are low, below the detection limit of laboratory methods.

#### 4.2.3. Profile C—Biological Pond Outflow

Surface water at the biological pond outflow has a lower content of ammonium ions (in comparison with the water quality at the drainage outflow), their content usually reaches a concentration up to 1 mg/L, rarely up to 3.85 mg/L, nitrites are low up to 0.1 mg/L, nitrates values are up to 10 mg/L. Specific conductivity reaches medium values, with the maximum of 64 mS/m. Water reaction is neutral to slightly alkaline (pH = 7.14 to 7.84). Heavy metals are low, in the case of Pb and Cd, they are below the detection limit of a laboratory method in all cases, while mercury reaches a maximum concentration of 0.0002 mg/L. COD and BOD values are often higher, with COD maximum concentration of 54.7 mg/L, in the case of BOD, it is 20.1 mg/L. PAH content reaches a maximum of 170 mg/L, the content of HOI is typically up to 0.5 mg/L.

#### 4.2.4. Profile D—Inflow into the Hamerský Stream

Surface water at the inlet to the Hamerský Stream does not show any signs of being affected by the leachate from the landfill; it is only affected by anthropogenous activity in the surroundings (agricultural areas), which is already apparent at the inlet profile. Surface water has low specific conductivity (up to 35 mS/m), with neutral to slightly alkaline water reaction (pH = 7.2–7.5). The contents of nitrogen substances are similar to the input profile. The maximum contents of ammonium ions are between 0.5 and 1.0 mg/L, the nitrite contents are up to 0.06 mg/L, the maximum nitrate contents are 41 mg/L. Maximum COD and BOD values reach 51.3 mg/L and 16.4 mg/L. Heavy metals are below the limit of detection, the maximum mercury content was 0.0001 mg/L. The concentration of PAH was, apart from one rare case where the concentration of PAH in April 2011 amounted to 340 mg/L, below the limit of detection by means of laboratory methods, HOI ranged up to 0.15 mg/L.

### 4.3. Discussion of the Results

Higher contents of ammonium ions, organic matter (COD, BOD) and polyaromatic hydrocarbons, as well as sporadic contents of HOI oil products, are irregularly monitored in leachate from the old reclaimed landfill. Based on the available results, landfill leachate is not a source of increased, above-limit contents of Hg and cannot therefore be considered as a contamination arising from this source.

**Figure 5:**
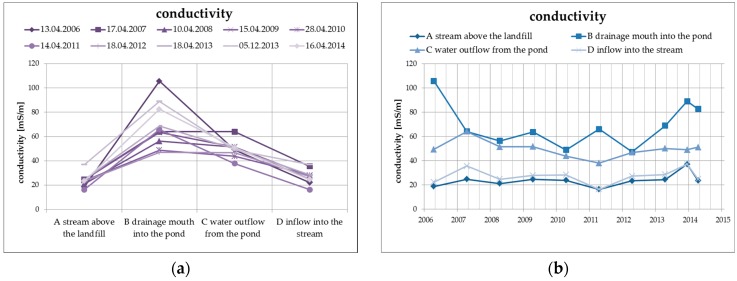
Change of values of specific conductivity in surface water depending on the monitored profile (**a**) and time (**b**).

The elimination of the negative properties of the leachate, including in particular the increased concentration of dissolved solid substances, ammonium ions, chlorides, and organic materials, takes place as a result of the physical and biogeochemical processes in the biological pond, where the leachate from the landfill is discharged. The primary process is the dilution of the concentrations of the main soluble substances, which is documented by the following Figure 5 presenting the specific conductivity values of surface water at the individual monitored profiles, including the long-term trend.

There is an average decrease of the values of specific conductivity by approximately 20%, mainly as a result of dilution and, from the long-term perspective, the trend is decreasing. The specific conductivity values at the inlet profile and at the outlet profile into the Hamerský Stream clearly show a balanced long-term trend with almost identical levels of specific conductivity values.

**Figure 6:**
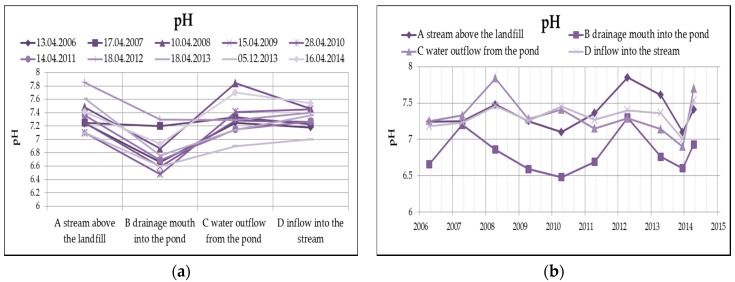
Change of values of pH in surface water depending on the monitored profile (**a**) and time (**b**).

The water reaction (pH) from Figure 6 clearly shows that the leachate has a slightly acidic reaction, after the outflow from the pond, the pH is already rather alkaline, which in this case is mainly caused by the dissolution of oxygen in water, respectively the reduction of oxygen, according to the following equation.

½O_2_ + 2e^−^ + 2H^+^ → H_2_O (1)

**Figure 7:**
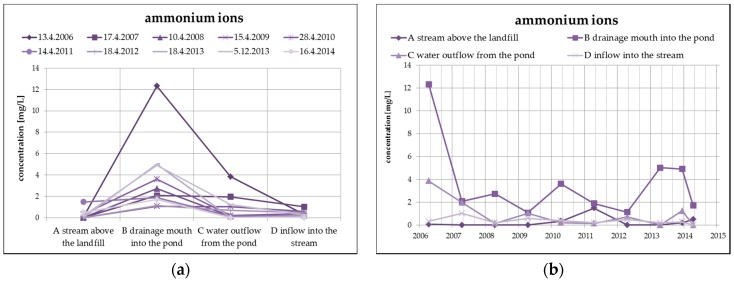
Change of values of ammonium ions in surface water depending on the monitored profile (**a**) and time (**b**).

The changes of the concentration of ammonium ions, typical representatives of landfill contaminants, take place mainly due to nitrification in the oxidizing environment (according to the following equation), which is evident from the following Figure 7. The highest concentration of ammonium ions in surface water from the monitored number of profiles is found in profile B (leachate outflow), while the concentration of nitrates is the lowest. However, a reduced concentration of ammonium ions with a simultaneous increase of nitrates can be observed at the outlet of the pond (profile C).

NH_4_^+^ + 2O_2_ → NO_3_^−^ + 2H^+^ + H_2_O (2)

**Figure 8:**
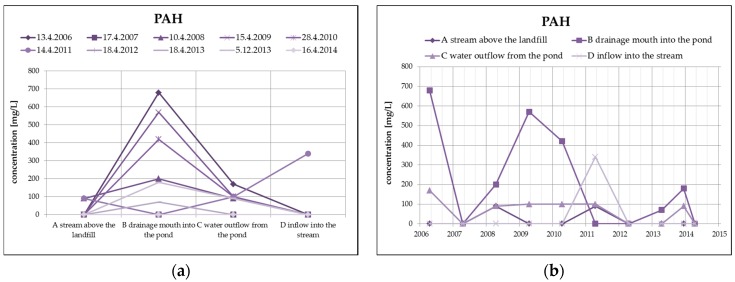

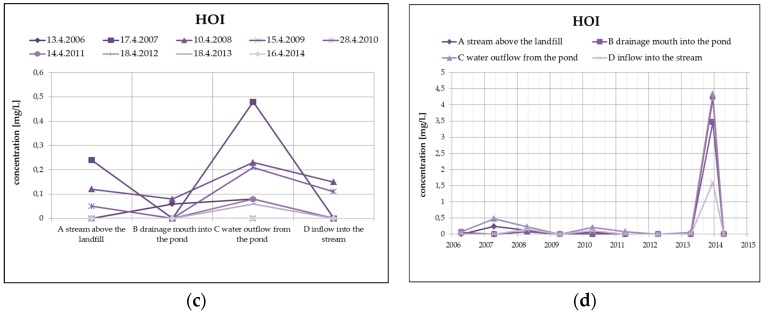
Change of PAH values in surface water depending on the monitored profile (**a**) and time (**b**) and change of HOI values in surface water depending on the monitored profile (**c**) and time (**d**).

From organic substances, there is an evident substantial decrease of PAH (by dilution), as well as a clear increase of HOI in surface water at the outlet of the pond, as you can see in Figure 8. HOI increase may in this case be caused by surface runoff from the surrounding areas as a result of the precipitation totals (secondary pollution without the impact of landfill seepages). From a long-term perspective, both monitored substances show a decreasing trend. The COD/BOD ratio indicates a prevalence of chemically degradable substances in comparison with biodegradable substances, and it ranges from 3.5 to 4 in the overall monitored set of profiles.

## 5. Conclusions 

The results of a long-term monitoring of ground, surface, and leachate water in the area of interest do not clearly show any relevant burden of the surrounding environment having a long-term and serious effect on the current level of quality of the Hamerský Stream and, respectively, its unnamed right-hand tributary. The potential source of pollution—Hamra landfill—has currently been reclaimed, surface-sealed, and planted. Leachate from the internal drainage system and rainwater from the peripheral drainage system are discharged into the biological pond, which functions as a mean of natural degradation of pollutants. A comparison of the quality of surface water at the inflow and outflow profile cannot unambiguously confirm the negative effect of the landfill body, the water quality is comparable. There are other anthropogenic activities in the area of interest that decrease the quality of surface water (agricultural activity in the vicinity), and there is also the possibility of increased geochemical background, especially in the form of mercury contents.

The main benefit of the biological pond is the dilution of the dominant contaminants, especially of inorganic nature, while ammonium ions show obvious nitrification as a result of their transition into an oxidizing environment. A long-term disadvantage of the natural degradation of leachate can be seen in the gradual decrease in efficiency due to higher concentration of the substances or the undesirable growth of aquatic plants; however, this can be effectively eliminated, e.g., by targeted aeration and by maintaining vegetation in the pond and its surroundings.

It can be concluded that the efficiency of the natural biochemical cleaning elements can be considered sufficient, taking into account the nature of the deposited waste, the amount and quality of leachate and, last but not least, also the climatic character of the locality.

## Figures and Tables

**Table 1:** Overview of the methodology of determination of the individual sample parameters, including the determination limit.

Parameter	Method	LOQ	Unit
BSK_5_	Oximeter (after 5 days)	3.8	mg/L
CHSK	Dichromate, spectrophotometry	3.8	mg/L
Ammonium ions NH_4_^+^	Spectrophotometry	0.13 (0.02) *	mg/L
nitrates NO_3_^−^	Spectrophotometry	0.4	mg/L
nitrites NO_2_^−^	Spectrophotometry	0.005 (0.006) *	mg/L
Mercury (Hg)	Fluorescence spectrometry	0.0001 (0.0005; 0.00005) *	mg/L
Other metals (Cd, Pb, Zn, As)	Emission spectrometry with inductively bound plasma	0.0001	mg/L
PAU	Gas chromatography	0.00006 (0.00009) *	mg/L
NEL	Infrared spectrometry	0.05	mg/L
pH	Potentiometric in situ using Cyberscan device	measurement range 7–14	-
Electrical conductivity	Potentiometric in situ using Cyberscan device	0.1	mS/m

Notes: * in the case of a different determination of the detection limit, it is caused by changing the device settings before the analysis in relation to the quality of the sample according to the decision of a laboratory technician.

**Table 2:** Overview of selected results of groundwater quality monitoring during the period of 2006–2014 (the determination limit values of LOQ are presented in Table 1).

Location	Date	Conductivity mS/m	pH	NH_4_^+^ mg/L	NO_2_^−^ mg/L	NO_3_^−^ mg/L	Hg mg/L	BOD mg/L
V-1	13.04.2006	36.80	7.08	<0.02	0.016	56.0	0.0014	10.10
	17.04.2007	29.00	6.51	<0.13	0.020	56.2	<0.0001	<3.80
	10.04.2008	29.40	6.93	<0.13	0.013	25.2	0.0001	4.50
	15.04.2009	17.80	7.30	<0.13	0.052	27.4	0.0001	13.80
	28.04.2010	20.90	6.99	<0.13	0.019	40.0	0.0009	6.10
	14.04.2011	14.50	7.02	<0.13	0.007	17.2	<0.0001	19.40
	18.04.2012	24.00	7.05	<0.13	0.016	33.5	0.0001	14.00
	16.04.2014	22.70	6.82	0.85	0.071	15.7	<0.00005	25.20
V-2	13.04.2006	29.70	7.09	0.04	0.023	34.0	<0.0001	16.60
	17.04.2007	54.50	6.84	0.4	0.028	10.1	<0.0001	20.40
	10.04.2008	32.30	7.14	<0.13	0.073	7.0	0.0003	25.60
	15.04.2009	26.30	7.13	<0.13	0.089	10.3	0.0002	18.40
	28.04.2010	29.90	6.93	<0.13	0.013	28.2	<0.0001	5.20
	14.04.2011	44.10	6.89	0.66	0.012	3.0	<0.0001	30.00
	18.04.2012	38.90	6.98	<0.13	0.013	19.0	<0.00005	19.90
	16.04.2014	48.40	6.25	0.89	0.044	5.8	<0.00005	26.40

**Table 3:** Overview of selected results of surface water quality monitoring during the period of 2006–2014.

Location	Date	Conductivity mS/m	pH	NH_4_^+^ mg/L	NO_2_^−^ mg/L	NO_3_^−^ mg/L	Hg mg/L	BOD mg/L	COD mg/L
A stream above the landfill	13.04.2006	18.70	7.24	0.06	0.046	22.00	0.0001	13.20	1.70
	17.04.2007	24.70	7.25	<0.13	0.020	6.00	0.0001	5.60	1.80
	10.04.2008	21.00	7.48	<0.13	0.032	14.40	0.0001	14.60	4.40
	15.04.2009	24.50	7.25	<0.13	0.027	8.30	0.0002	12.40	6.00
	28.04.2010	23.70	7.10	0.33	0.023	7.50	<0.0001	7.60	2.50
	14.04.2011	16.30	7.36	1.48	0.020	13.30	0.0001	44.20	11.60
	18.04.2012	23.30	7.85	<0.13	0.009	4.10	0.0002	14.10	2.20
	18.04.2013	24.40	7.61	<0.13	0.013	37.20	0.0001	84.00	22.30
	05.12.2013	37.00	7.10	0.19	0.076	71.30	<0.0005	20.50	1.00
	16.04.2014	23.50	7.41	0.49	0.052	13.10	<0.00005	53.00	19.50
B drainage mouth into the pond	13.04.2006	105.70	6.66	12.32	0.030	2.00	<0.0001	43.80	14.50
	17.04.2007	64.00	7.20	2.08	0.090	2.40	0.0001	29.80	5.00
	10.04.2008	56.30	6.86	2.72	0.044	1.10	<0.0001	17.90	5.30
	15.04.2009	63.50	6.59	1.08	0.025	1.40	0.0002	22.10	10.00
	28.04.2010	48.80	6.48	3.60	0.005	0.70	<0.0001	12.30	4.80
	14.04.2011	65.90	6.69	1.88	0.006	4.10	<0.0001	16.00	1.80
	18.04.2012	47.10	7.30	1.12	0.014	0.40	0.0001	36.40	21.80
	18.04.2013	68.80	6.76	5.02	0.061	7.20	0.0001	31.80	6.00
	05.12.2013	89.00	6.60	4.91	0.049	2.60	<0.0005	26.50	4.00
	16.04.2014	82.50	6.93	1.70	0.025	5.10	<0.00005	27.10	8.00
C water outflow from the pond	13.04.2006	49.20	7.25	3.85	0.021	8.00	0.0001	23.20	3.70
	17.04.2007	64.00	7.33	1.96	0.096	2.30	0.0001	27.80	6.50
	10.04.2008	51.30	7.84	0.14	<0.006	1.40	0.0001	31.80	9.00
	15.04.2009	51.50	7.28	1.03	0.120	4.10	0.0002	25.20	12.70
	28.04.2010	43.80	7.41	0.21	0.024	1.20	<0.0001	19.10	7.50
	14.04.2011	37.90	7.15	0.14	<0.005	<0.40	<0.0001	36.40	9.00
	18.04.2012	46.70	7.29	0.69	0.010	<0.40	0.0001	32.20	7.20
	18.04.2013	50.00	7.14	<0.13	0.084	10.20	0.0001	34.00	7.30
	05.12.2013	49.00	6.90	1.23	1.471	12.80	<0.0005	39.00	4.00
	16.04.2014	51.00	7.70	<0.13	0.027	<0.40	<0.00005	54.70	20.10
D inflow into the stream	13.04.2006	22.20	7.18	0.32	0.036	25.00	<0.0001	12.60	1.20
	17.04.2007	35.50	7.23	1.02	0.060	12.30	<0.0001	10.00	1.40
	10.04.2008	24.70	7.46	0.20	0.031	14.30	<0.0001	13.10	4.20
	15.04.2009	27.70	7.26	0.59	0.039	10.20	0.0001	9.00	4.00
	28.04.2010	28.20	7.45	0.40	0.032	8.10	<0.0001	8.10	3.00
	14.04.2011	16.30	7.27	0.19	0.021	13.90	0.0001	41.30	9.20
	18.04.2012	27.20	7.40	0.49	0.019	3.70	0.0001	16.00	1.30
	18.04.2013	28.50	7.36	0.18	0.022	40.70	0.0001	17.60	5.00
	05.12.2013	37.00	7.00	0.27	0.066	66.00	<0.0005	18.80	1.00
	16.04.2014	24.60	7.54	<0.13	0.059	12.70	<0.00005	51.30	16.40
